# The Incidence of Central Serous Chorioretinopathy after Photorefractive Keratectomy and Laser In Situ Keratomileusis

**DOI:** 10.1155/2012/904215

**Published:** 2012-02-12

**Authors:** Majid Moshirfar, Maylon Hsu, Julia Schulman, Joseph Armenia, Shameema Sikder, M. Elizabeth Hartnett

**Affiliations:** ^1^John A. Moran Eye Center, University of Utah Salt Lake City, UT 84121, USA; ^2^School of Medicine and Biomedical Sciences and Ira G. Ross Eye Institute, University at Buffalo, State University of New York, Buffalo, NY 4260, USA; ^3^Wilmer Eye Institute, Johns Hopkins University, Baltimore, MD 21287, USA

## Abstract

*Purpose*. To assess the incidence of central serous chorioretinopathy (CSCR) following laser in situ keratomileusis (LASIK) and photorefractive keratectomy (PRK). *Methods*. A chart review was performed to identify all patients with CSCR and a previous history of LASIK or PRK. *Results*. Over the 6-year study period, 1 of 4,876 eyes which had LASIK or PRK at the Moran Eye Center was diagnosed with CSCR. One other patient was referred from an outside center, developed CSCR symptoms one month after PRK. Both patients were managed conservatively with a final visual acuity of 20/20 or better. All other patients presented 4 or more years after refractive surgery. *Conclusions*. We report the first 2 CSCR cases developing within one month after PRK. The low incidence argues against a causal association. Topical corticosteroids or anxiety may elevate cortisol levels presenting therapeutic challenges for the management of CSCR after PRK or LASIK.

## 1. Introduction

Vitreoretinal pathology has rarely been observed after laser-assisted in situ keratomileusis (LASIK) or photorefractive keratectomy (PRK) [1]. Posterior segment complications are more common in myopic eyes and no direct causal relationship with keratorefractive surgery has been established [2]. Retinal breaks, rhegmatogenous detachments, posterior vitreous detachments, choroidal neovascularization (CNV), and vitreous hemorrhage have all been reported [1–3].

Central serous chorioretinopathy (CSCR) is generally a self-limited disease with the majority of patients recovering visual acuity to 20/30 or better within three months [4]. Approximately 4% of patients will present with binocular involvement, while up to 40% may eventually have involvement of the fellow eye [5]. Increased permeability of the choroidal vessels and dysfunction of the retinal pigment epithelium (RPE) have been observed with neurosensory retinal detachment and pigment epithelial detachment. Chronic cases of CSCR may develop RPE atrophy and rarely CNV [6]. Patients present with central visual aberrations including scotoma, metamorphopsia, dyschromatopsia, and micropsia [7]. Known risk factors for CSCR include “type A” personality, pregnancy, and corticosteroid elevation either endogenously or through exogenous corticosteroid use [8].

CSCR has been reported in 4 separate case reports after LASIK [9–12]. To date, there have not been any reports of CSCR after PRK. The goal of this study was to report the incidence of CSCR after both PRK and LASIK through a retrospective chart review.

## 2. Methods and Patients

From January 2005 to December 2010, an ICD-9 code search for CSCR (362.41) identified 250 CSCR patients seen at the John A. Moran Eye Center. All patients had a comprehensive evaluation by a retina specialist. A retrospective chart review identified 7 patients with a prior history of refractive surgery. Patients' age, sex, medication history, pre- and postrefractive surgery exam findings, onset of CSCR symptoms, clinical course, treatment, and visual outcomes were analyzed. The University of Utah Hospital Institutional Review Board approved the research protocol in accordance with the tenets of the Declaration of Helsinki.

Database billing records of the Moran Eye Center were reviewed to determine the total number of LASIK and PRK procedures performed during the 6-year study period. Prior to 2008, a Hansatome microkeratome (Bausch & Lomb, Inc, Rochester, NY) was used for flap creation. Thereafter, the IntraLase FS60 femtosecond laser (Abbott Medical Optics (AMO), Santa Ana, CA) was utilized. Both PRK and LASIK were performed with either the VISX Star S4 CustomVue (AMO), LadarVision CustomCornea (Alcon Inc., Fort Worth, TX), or the WaveLight Allegretto (Alcon, Inc.) excimer laser platforms.

 All Moran Eye Center refractive surgery patients are routinely followed at a minimum of 6 visits at 1 day, 1 week, 1, 3, 6, and 12 months after surgery. Any retinal complications that are discovered during postoperative exams are routinely referred to our institution's retina service. Because of our internal referral system, any CSCR complication after LASIK or PRK would be identified in the review of retina record visits.

A MEDLINE keyword search for CSCR and PRK or LASIK identified all previously reported cases of CSCR after LASIK or PRK in the literature.

## 3. Results

Of the 250 CSCR patients seen by the retina service, 7 patients were identified as having a previous history of LASIK or PRK at either the Moran Eye Center or an outside facility (Table 1). All 7 patients were male with an average age of 37 years, and all presented with unilateral CSCR. All had myopic keratorefractive treatment, and none had a previous history of CSCR or documented oral, topical skin, or inhaled corticosteroid use or other significant medical history.

Only 2 patients (Patients 1 and 2) had onset of disease within the immediate postoperative period of 1 month. Details of their clinical courses are described below. The remaining patients presented more than 4 years after refractive surgery and were not considered to have a potential causal relationship. All 7 patients were followed at the Moran Eye Center from the time of initial CSCR symptoms to resolution or stabilization of disease. Two of the patients received treatment with focal laser photocoagulation, while 5 resolved spontaneously without treatment. All 7 patients improved to an uncorrected distance visual acuity (UDVA) of 20/30 or better. Best corrected visual acuity (BCVA) was not recorded in all patients.

From January 2005 to December 2010, there were 2,728 eyes which had LASIK, and 2,148 eyes that received PRK at the Moran Eye Center. Only one of the combined 4,876 total LASIK and PRK cases at our institution developed CSCR within one month. The annual incidence of CSCR over the 6-year study period was calculated to be 3.4 per 100,000 eyes for both PRK and LASIK cases, and 7.8 per 100,000 eyes of only PRK cases.

A comprehensive literature search revealed 4 other reports of CSCR after LASIK; however, long-term recovery was not available in all cases (Table 1). Two patients presented with bilateral CSCR [10, 11] and 1 patient developed a rapid onset of CSCR with CNV one week following LASIK [12].

## 4. Patient 1

This is a 37-year-old male with an unknown employment who underwent PRK in September 2010 with a preoperative manifest refraction of −4.50 + 0.25 × 134 and −4.50 sphere, right and left eyes, respectively. Dilated fundus exam noted nonspecific RPE clumping in the left macula. On postoperative day 1, UDVA was 20/20 in both eyes. The patient was started on prednisolone acetate 1% (Allergan Inc., Irvine, CA), 4 times a day for one month. Nineteen days after PRK, the patient reported central metamorphopsia in his left eye. BCVA was 20/40 in the left eye with manifest refraction of −0.50 sphere. Anterior segment exam showed clear corneas without epithelial defects or stromal haze.

**Figure 1:**
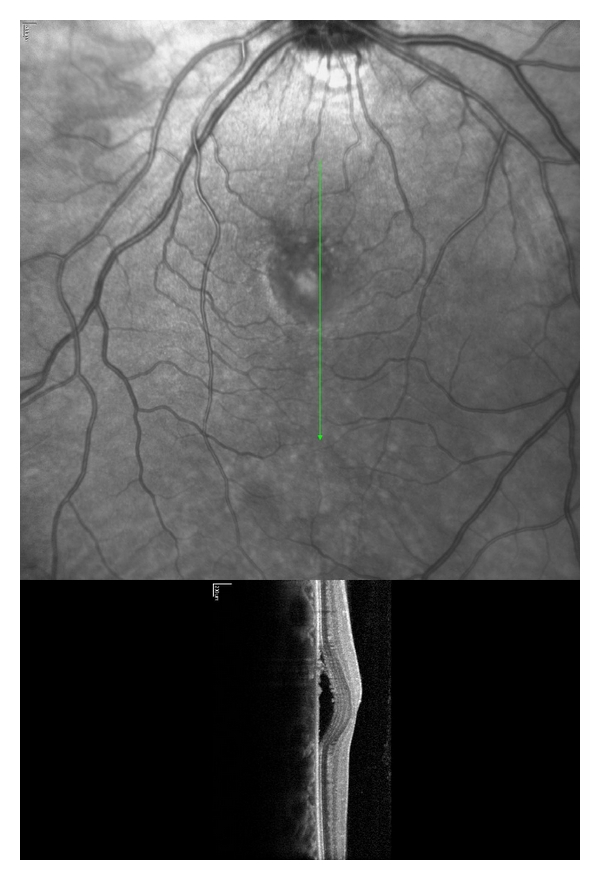
Fundus photograph and optical coherence tomography of the left eye of Patient 1, 19 days after PRK, showing serous retinal detachment consistent with CSCR.

Spectral domain optical coherence tomography (sd-OCT, Heidelberg Engineering, Heidelberg, Germany) demonstrated subretinal fluid accumulation consistent with CSCR (Figure 1) in the affected eye. No abnormalities were seen in the right eye. Consultation with a retina specialist led to the decision to keep the patient on standard corticosteroids drops with close observation. After one month, the patient was switched to fluorometholone 0.1% (Allergan Inc.) 4 times daily which was tapered over the next 6 weeks. His total postoperative corticosteroid exposure was 12 weeks.

 UDVA improved to 20/20 bilaterally 1 month after initial symptoms with complete resolution of metamorphopsia by 5 months after PRK. Eight months after PRK, UDVA was 20/20 in both eyes. The patient reported a mild recurrence of central visual distortion in the left eye. Anterior and posterior segment exam and sd-OCT findings were normal and the patient was advised to monitor his symptoms without further intervention.

## 5. Patient 2

This is a 28-year-old male lab technician who underwent PRK at an outside facility in August 2009. His preoperative manifest refraction was −4.50 + 1.00 × 90 and −4.75 + 1.75 × 90, right and left eyes, respectively. He began experiencing central metamorphopsia in the left eye 1 month after surgery. He was referred to our center 3 months later. His vision at that time was 20/20 in both eyes. The diagnosis of CSCR was confirmed by fundus exam and OCT subretinal fluid in the left eye, with a normal exam in the right eye. His postoperative medications were not recorded. This patient was followed until spontaneous resolution of his symptoms. Ten months after PRK his UDVA was 20/15 in the left eye with no visual complaints.

## 6. Discussion

CSCR as a complication of refractive surgery is rare with only 4 previously reported cases [9–12]. These reports identified 2 hyperopic [9, 10] and 1 myopic patient [11] who developed CSCR shortly after LASIK and 1 patient [12] who developed CSCR with CNV. We describe the first reported cases of CSCR to present after PRK.

The annual age-adjusted incidence of CSCR has been reported to be 5.8 per 100,000, with a male incidence of 9.9 and a female incidence of 1.7 per 100,000 [4]. Although not directly comparable with population studies, our findings support a large male predominance with a low annual incidence of 7.8 per 100,000 eyes after PRK and 3.4 per 100,000 eyes after both PRK and LASIK, based on analysis of the cases performed at our institution over 6 years. There were 5 patients who developed CSCR 4 years or more after LASIK or PRK. These patients were excluded from our analysis as having CSCR potentially caused by LASIK or PRK. Limitations in our study include the retrospective nature, possible loss of followup, and development of CSCR after the time period of medical chart review.

Acute CSCR is a self-resolving disease in approximately 90% of cases [5]. Chronic CSCR often is bilateral, with symptoms lasting longer than 6 months and can occur with recurrent disease. CNV has been reported in 0.3–2% of cases [7]. The high percentage of spontaneously resolving cases is the reason conservative management is initially advised. Corticosteroids should be discontinued and if resolution is not observed within 3 months more aggressive management can be considered. Half-fluence photodynamic therapy (PDT) and photocoagulation result in more rapid resolution of subretinal fluid, and may also limit recurrences [7].

Previous studies that explored processes leading to vitreoretinal pathology following refractive surgery have identified two possible exposures. The first is potential shock wave damage in the posterior segment during excimer laser treatment [1]. This exposure is shared by LASIK and PRK. However, Krueger et al. reported insignificant retinal stress wave amplitudes in experimental models after excimer photoablation [13]. The second potential risk during LASIK is the rapid change in intraocular pressure from the suction ring used to stabilize the microtome [1]. Although less vacuum is required with femtosecond-laser-assisted LASIK, the longer flap creation time may still predispose patients to retinal pathology [2]. Both mechanisms can theoretically destabilize a structurally altered retina such as in the case of CSCR.

Corticosteroids are often used after PRK for their anti-inflammatory properties as an attempt to prevent long-term sequelae of haze and unpredictable refractive outcomes [14]. Although, some reports have shown no difference in outcomes following PRK treated with or without topical corticosteroids [15, 16], most PRK surgeons reported using some form of topical corticosteroids postoperatively [17]. Early corticosteroid treatment has also been reported more effective than nonsteroidal anti-inflammatory drugs [18].

Multiple routes of corticosteroid administration [19] have been associated with CSCR, including oral, intravenous, epidural, intra-articular, inhaled, topical skin application, and also following intravitreal triamcinolone injections [20]. However, to our knowledge there have been no reported cases of CSCR following topical ocular corticosteroid use. The lack of such cases may reflect low penetration of topical drugs in reaching the posterior segment, with even lower systemic absorption due to further dilution in the tear film [21]. In rabbit eyes, difluprednate showed dramatically decreased concentrations in the posterior retina and barely detectable blood concentrations [22], suggesting that even a highly lipophilic topical corticosteroid with excellent corneal penetration may not significantly alter retinal pathology. Alternatively, the stress and anxiety of refractive surgery inducing elevated levels of systemic cortisol may contribute to exacerbations in CSCR patients.

The dilemma for the surgeon is how to treat such a patient who develops CSCR after PRK. Continuing topical corticosteroids may prolong or exacerbate CSCR symptoms, whereas early removal may decrease control over inflammation potentially leading to long-term haze. Unfortunately, there is no strong evidence in favor of either management strategy. Whether topical corticosteroids are discontinued or changed to less potent formulations, worsening CSCR may still require consideration of PDT or laser photocoagulation.

The decision was made to continue our patient on topical corticosteroids with close clinical observation. The subretinal fluid and symptoms resolved, with no postoperative corneal haze. The patient was noted to have mild RPE changes preoperatively which may have indicated prior CSCR disease activity. A dilated fundoscopic exam is an important part of both the refractive screening process and postoperative evaluation when outcomes are not as expected.

In summary, from review of the literature and analysis of over 4,800 consecutive LASIK and PRK cases at our institution, we report a low incidence of CSCR after LASIK or PRK without a direct causal association. CSCR is a multifactorial disease with an overall good prognosis. CSCR after keratorefractive surgery may rarely be induced by extensive topical corticosteroids, elevated cortisol levels from stress of the surgery, or mechanical disturbances after excimer laser or LASIK flap creation in susceptible patients.

## Figures and Tables

**Table 1:** Baseline and clinical data of CSCR patients with a history of LASIK or PRK.

Patient	Gender; age	LASIK or PRK	Pre-LASIK or PRK Manifest Refraction	Location of surgery	Time of CSCR onset after LASIK or PRK	CSCR involv- ement	CSCR therapy	UDVA at onset of CSCR	BCVA at onset of CSCR	Final UDVA	Final BCVA
1	M; 37	PRK	−4.50 sphere	Moran	19 days	OS	Observe	20/40	20/40	20/20	20/20
2	M; 28	PRK	**−**4.75 + 1.75 × 90	Outside	1 month	OS	Observe	20/20	20/20	20/15	Unknown
Wang et al. [7]	M; 50	LASIK	+0.5 − 1.25 × 155	Singapore	1 week	OD	Laser	Unknown	20/40	20/20	20/20
Haimovici et al. [8]	M; 54	LASIK	**−**7.00 − 1.00 × 180 ** −**6.75 − 1.25 × 175	Greece	1 month	OU	PDT	Unknown	20/50 20/50	Unknown	20/30 20/25
Lim et al. [9]	M; 33	LASIK	+4.75 − 0.75 × 160 +6.25 − 2.00 × 180	India	4 days	OU	Observe	20/200 20/125	20/125 20/50	Unknown	20/30 20/30
Sighvi et al. [10]	F; 20	LASIK	**−**7.00 + 1.00 × 90	Saudi Arabia	1 week	OD	PDT	Unknown	20/200	Unknown	20/60

M: Male; F: Female; PRK: Photoreactive Keratectomy; LASIK: Laser-assisted in situ keratomileusis; PDT: Photodynamic Therapy; Laser: Focal Photocoagulation; OD: Right Eye; OS: Left Eye; OU: Both Eyes; UDVA: Uncorrected distance visual acuity, BCVA: Best corrected visual acuity. Final UDVA and BCVA were from the last recorded follow-up visit.
